# The Relation between Parental Locus of Control and Willingness to Implement Parent Management Training Strategies

**DOI:** 10.1007/s10578-024-01748-5

**Published:** 2024-08-16

**Authors:** Catherine E. Drott, Sara R. Elkins, Tessa K. Kritikos

**Affiliations:** 1 University of Houston Clear Lake, Houston, TX USA; 2 University of Houston, Tilman J. Fertitta Family College of Medicine, Houston, TX USA; 3 Loyola University Chicago, Chicago, IL USA

**Keywords:** Parenting, Disruptive behavior, Parent management training, Parental locus of control

## Abstract

In child disruptive behavior treatment, successful parent management training (PMT) outcomes are dependent on parents’ use of strategies outside of sessions. This study aimed to identify the influence of parental locus of control (PLOC) on a key treatment acceptability variable: parents’ willingness to implement PMT strategies. We sought to investigate this relationship for individual strategies within PMT, given the composite nature of the intervention. In this study, 109 parents of children (ages 2–12) with disruptive behavior watched brief videos detailing three proactive PMT strategies (child-directed interaction, effective commands, positive attention) and three reactive PMT strategies (ignoring, time out, and removal of privileges) and rated their willingness to implement each strategy. Internal PLOC predicted greater overall willingness to use PMT strategies, above and beyond the influence of child age, child gender, and disruptive behavior severity. Notably, the relationship between willingness and PLOC differed across individual strategies. PLOC predicted willingness to implement proactive PMT strategies to a greater degree than willingness to implement reactive strategies. External PLOC may be a greater barrier to use of proactive strategies because of these strategies’ misalignment with external PLOC-related beliefs. Results have implications for the personalization of PMT based on parent cognitions, as well as for the separate analysis of individual components of PMT in future research.

## Introduction

Parent management training (PMT) is an evidence-based treatment for disruptive behaviors that often occur in neurodevelopmental, impulse-control, and conduct disorders, or in the absence of a specific disorder [1–3]. Starting in early childhood, many disruptive behaviors are maintained through a coercive cycle (e.g., Patterson, [4, 5] in which the child’s behavior both elicits and reinforces ineffective parent behaviors. Concurrently, the parent’s behavior elicits and reinforces the child’s disruptive behaviors, which may include noncompliance, aggression, elopement, or tantrums. The significant influence of parent behavior on child behavior necessitates parent involvement in treatment. PMT seeks to train parents to prevent and respond appropriately to disruptive behavior, thereby reducing parent elicitation and reinforcement of the behavior and halting the coercive cycle [2, 6].

There is stronger empirical support for the effectiveness of PMT than for any other child disruptive behavior intervention, including medication or other forms of therapy [2]. PMT is also an adaptable intervention, as it can be presented in individual or group settings, with the child and parent or parent alone, and in face-to-face or virtual venues [2]. However, low treatment engagement, which includes both attendance and parental compliance with interventions, is a widespread problem and a significant barrier to treatment success [7–9]. One review of 262 studies found approximately 25% of parents referred for PMT never enroll in treatment and an additional 26% attend at least one session but drop out before completing the intervention [10]. Attrition rates for studies measuring PMT effectiveness, suggesting similar conditions to community samples, were commensurate with efficacy studies with higher degrees of control [10]. High attrition rates were, however, associated with low SES [10]. When parents do attend sessions, quality participation, such as active involvement during in-session activities and completion of homework, is necessary for improvements in child behavior and varies widely [10, 11]. Low engagement rates highlight the need to understand influencers of parental engagement in PMT.

There are many predictors of parental engagement in PMT identified in the literature. Such influences range from systemic and structural elements (i.e., cost, transportation, mode of delivery**)** to parent and family characteristics (i.e. parent stress, mental health, culture, religion) [11, 12]. These factors can be viewed as barriers to engagement, as well as opportunities for adaptation and intervention. There is increasing attention in the literature to parent cognitions as predictors of PMT engagement and child behavior outcomes, as opposed to demographic characteristics like marital status or socioeconomic status [8, 13]. Parent cognitions are particularly relevant in the parent’s initial acceptance of and engagement in PMT [14]. These cognitive predictors offer an individualized understanding of each parent’s internal barriers to treatment. For example, treatment engagement and outcomes are influenced by parents’ beliefs about the cause of the child’s behavior, their expectations for treatment, and their own parenting abilities [15, 13]. Clinicians can use this information to personalize PMT treatment plans [16, 17]. For example, parents with maladaptive treatment-related cognitions may benefit from extra efforts to build rapport, supplemental psychoeducation, or a change in treatment format (e.g. group vs. individual).

Among the internal parent characteristics pertinent to PMT is parental locus of control (PLOC), a set of beliefs about parental self-efficacy and control of child behavior [18]. Parents with an internal PLOC generally believe their parenting practices influence their child’s behavior. Conversely, parents with an external PLOC are likely to attribute their child’s behavior to factors such as child characteristics, situational causes, or chance. Greater externality of PLOC is observed in parents of children with disruptive behavior than parents of children without disruptive behavior [19]. Parents with an external PLOC are more likely to use ineffective parenting practices, ranging from overreliance on punitive discipline to lax or permissive parenting styles [20, 21]. Such parents tend to believe their parenting behavior is unlikely to change their child’s behavior [20]. Parents who have low parental self-efficacy, suggesting external PLOC, may view treatment as unnecessary or unlikely to help, leading to reduced engagement in treatment [15]. Similarly, Lawton et al. [22] found parents with low parental self-efficacy and a higher belief in fate and chance were likely to believe symptoms would resolve without treatment.

PLOC is thought to be an important parental factor in the personalization of PMT because parent training interventions are based on the assumption that parents can change their child’s behavior by modifying their parenting practices. This underlying premise is, in many ways, incompatible with an external PLOC. Miller and Prinz [23] investigated the relationship between parental attributions of their child’s behavior and treatment engagement in PMT and found parents were most engaged and successful when the initial treatment matched their attributions of the child’s behavior and need for intervention. For example, parents who attributed the child’s behavior problems to the child’s weaknesses (i.e. anger, lack of self-control), suggesting an external PLOC, were less likely to remain engaged in treatment directed at parent skills, without child involvement in sessions. Parents who attributed their child’s disruptive behavior to their own parenting behaviors were more likely to continue with treatment focused on parent skills [23]. This finding highlights the importance of identifying the parents’ pre-treatment cognitions.

Another contributor to parent engagement in PMT, which relates to parents’ fidelity of implementation of strategies, is the acceptability of the intervention. Treatment acceptability refers to the client’s perception of whether the intervention is reasonable and appropriate for their situation [24]. Riemers et al. [25] conceptualize treatment acceptability as a 6-factor construct, reflecting the parent’s perception of the treatment’s reasonableness, effectiveness, disadvantages, and disruptiveness, as well as the expected time required to engage and the parent’s willingness to do so. Reimers et al. [26] found treatment acceptability is highest when the intrusiveness of the treatment is matched to the severity of the disruptive behavior. Medication (considered more intrusive) was rated as more acceptable for severe behaviors and positive reinforcement (less intrusive) was rated more acceptable for less severe behaviors. This variable relationship between acceptability and treatment type is particularly relevant for a composite intervention such as PMT. PMT programs teach parents a variety of distinct strategies, some of which are designed to build the parent-child relationship and proactively prevent disruptive behavior [2]. Other PMT strategies are for reactive use, to be employed by the parent when the disruptive behavior occurs [2]. Further, strategies differ in the amount of parent skill required. Strategies such as positive attending and praise are conceptually simple, while strategies like time out have many more steps and logistics. Given the variable relationship between treatment acceptability and treatment type, other parent cognitions, such as PLOC, may have a similarly variable relationship with the qualities of individual behavior management strategies.

Greater treatment acceptability is theorized to relate to better treatment outcomes. In a study of mothers of children with ODD, acceptability and child behavior improvement were positively correlated [27]. Similarly, Eyberg [28] noted parents who reported high levels of treatment acceptability experienced better maintenance of child behavior improvements over time. However, findings are mixed regarding the strength of the relationship between acceptability and outcomes [29]. Some have suggested there is a minimum level of acceptability required for treatment efficacy, rather than a continuous relationship between acceptability and outcome. In this view, a certain level of acceptability is crucial, but increases beyond the minimum threshold are insignificant [15]. Identifying the role of acceptability is complicated by the variation in construct definitions as well as inconsistent methods of measurement [30]. For the present study, we focus on willingness to implement, the component of acceptability most likely to impact a parent’s implementation of the intervention soon after it is introduced [25]. By identifying willingness to implement, we gain insight into the parent’s motivation to participate in treatment, as well as the likelihood of their engagement.

## Current Study

Parent cognitions, including PLOC, and the willingness to implement component of acceptability are independently established as likely contributors to parental engagement and child behavior outcomes [25, 31], but there is less inquiry into the relationship between the two. Current literature indicates a correlation between components of treatment acceptability and components of PLOC. For example, Johnston et al. [15] found greater parental self-efficacy was related to greater perception of treatment effectiveness. Chase & Peacock [29] investigated similar constructs and found external PLOC was related to lower acceptability of a written description of PMT. The purpose of this study is to investigate this relationship further and guide future explorations of the clinical implications of PLOC. A greater understanding of the relationship between PLOC and willingness to implement strategies could have implications for treatment personalization and related outcomes. In addition, as influencers of PLOC are better understood, findings could have implications for the assessment, diagnosis, and referral process, potentially impacting initial engagement in PMT after a referral.

### Hypotheses

First, it was hypothesized that PLOC would be correlated with overall willingness to implement PMT strategies, with an inverse relationship between externality of PLOC and willingness to implement strategies. Second, this study explored parent perceptions of individual components of PMT. This may provide insight into the areas within PMT that are most critical to modify or supplement for parents with an external PLOC. It was hypothesized that internal PLOC would predict willingness to implement proactive, or antecedent-based, strategies, while external PLOC would predict willingness to implement reactive, or consequence-based, strategies. This hypothesis was based on the finding that parents with an external PLOC are more likely to utilize an authoritarian or reactive parenting style [20, 21] and the premise that an external PLOC relates to a limited belief in the parent’s ability to prevent disruptive behavior. Finally, this study investigated the ability of PLOC to predict parental willingness to implement PMT strategies above and beyond the predictive power of the child’s level of disruptive behavior. Severity of disruptive behavior is related to PLOC (i.e., greater severity is linked to greater external PLOC; [32]), and severity is also linked to acceptance of parent training treatments (e.g., [29, 30]. It was therefore hypothesized that a significant percentage of the variance in willingness would be predicted by severity, but the addition of PLOC to the model would account for additional variance in willingness to implement.

## Methods

### Recruitment and Selection Criteria

Participants were recruited via flyer posted to an online forum for parents of children with ADHD, parent-focused social media sites, public schools, community organizations, and behavioral health clinics. The flyer called for caregivers of children ages 2 to 12 who displayed disruptive behaviors such as noncompliance, defiance, aggression, or tantrums. Participants were not excluded based on diagnosis or lack thereof. However, parents completed the Strengths and Difficulties Questionnaire (SDQ) to rate the severity of their child’s behavior. Participants who rated their child’s behavior as average on the Hyperactivity and Conduct Problems SDQ scales were excluded from the study, as they were unlikely to meet the disruptive behavior inclusion criteria. Those who reported prior participation in a disruptive behavior intervention (including Parent Management Training programs or Applied Behavior Analysis) were excluded to limit the possibility of prior exposure to strategies, which could impact acceptability.

### Participants

An a priori power analysis in G*Power [33] for bivariate correlation estimated between 88 and 131 participants (α = 0.05) were needed to achieve 80% power to detect small to medium effect sizes (i.e. *r* = .24–0.29; based on typical effect sizes comparing PLOC and treatment acceptability; Johnston et al. [15]. A sample of 122 participants was obtained. Four participants were removed due to incomplete data and nine participants were removed due to average SDQ Hyperactivity and Conduct Problems scores, resulting in a sample size of 109. The remaining participants were primarily female, biological parents. 65% of participants identified as Caucasian (white, not of Latino or African descent), 12% identified as bi-racial or multi-racial, 12% identified as Latino/a or Hispanic and 6% identified as Black, African America, or Caribbean. 60% of parents reported attaining a Bachelor’s degree or higher. The children the participants reported on were 63% male, with a mean age of 7.1 (*SD* = 2.8). 56% were reported to be Caucasian (white, not of Latino or African descent), 22% were bi-racial or multi-racial, 12% were Latino-a or Hispanic, 6% were Black, African America, or Caribbean, and 3% were Asian. 26% of caregivers reported their child did not have a psychological diagnosis and 71% of caregivers reported their child is diagnosed with ADHD. Additional detailed demographic information of participants and participants’ children is presented in Table 1 and Table 2, respectively.

**Table 1:** Sociodemographic characteristics of caregivers

Characteristic	*N* = 109	
	*n*	%
Caregiver Gender		
Female	95	87
Male	13	12
Gender non-binary	1	< 1
Caregiver Age		
25–34	31	28
35–44	51	46
45–54	25	23
55–64	2	2
Caregiver Type		
Biological Parent	102	93
Adoptive Parent	5	2
Step-Parent	1	< 1
Legal Guardian	1	< 1
Caregiver Race/Ethnicity		
American Indian or Alaskan Native	1	< 1
Asian	2	2
Black (African American or Caribbean)	7	6
Latino/a or Hispanic	13	12
Caucasian (White, Not of Latino or African Descent)	72	65
Middle Eastern or North African	1	< 1
Biracial or Multiracial	13	12
Caregiver First Language		
English	97	88
Mandarin Chinese	1	3
Spanish	6	3
Other	5	5
Caregiver Education		
High School Diploma	14	13
Specialized Trade or Technical Degree	8	5
Associates Degree	21	19
Bachelor’s Degree	41	37
Master’s Degree	22	20
Doctoral Degree	3	3
Household Income		
<$20,000	6	5
$20,000–40,000	19	17
$41,000–60,000	19	17
$61,000–80,000	12	11
$81,000-100,000	14	13
>$100,000	39	35
Parenting Status		
Single Parenting	15	13
Co-parenting in the same home	76	69
Co-parenting in separate homes	18	16

_Note. Percentage values were rounded to the nearest integer_

**Table 2:** Sociodemographic characteristics of children

Characteristic	*N* = 109	
	*n*	%
Child Gender		
Female	40	36
Male	69	63
Child Age		
2	1	< 1
3	6	6
4	8	7
5	8	7
6	12	11
7	7	6
8	11	10
9	17	16
10	15	14
11	8	7
12	16	15
Child Race/Ethnicity		
Asian	3	3
Black (African American or Caribbean)	6	6
Latino/a or Hispanic	13	12
Caucasian (White, Not of Latino or African Descent)	61	56
Middle Eastern or North African	1	< 1
Biracial or Multiracial	24	22
Child Diagnostic Status		
No Diagnosis	28	26
One Diagnosis	38	35
Multiple Diagnoses	43	39
Child Diagnoses, based on parent report		
Attention-Deficit/Hyperactivity Disorder	77	71
Oppositional Defiant Disorder	14	13
Autism Spectrum Disorder	8	7
Disruptive Mood Dysregulation Disorder	5	5
Anxiety	31	28
Depression	4	4
Other	6	6

_Note. Percentage values were rounded to the nearest integer_

### Measures

#### Demographic Questionnaire

Caregivers completed a questionnaire and provided information about their relationship to the child, parent and child’s age and gender, race and ethnicity, education and income level, and parenting relationship status. They also reported their child’s diagnostic status by selecting from a list of common childhood diagnoses (ADHD, ODD, ASD, DMDD, anxiety, depression), writing in “other” diagnoses, or selecting “no diagnosis.”

#### Child Symptoms Measure

Parents completed the Strengths and Difficulties Questionnaire (SDQ) to assess the child’s behavioral functioning [34]. The SDQ includes five subscales: Emotional Problems, Conduct Problems, Hyperactivity, Peer Problems, and Prosocial Behavior. The Hyperactivity and Conduct Problems scales were summed to create the SDQ Externalizing score, used as a continuous indicator of behavior severity. Based on a systematic literature review, confirmatory factor analysis supports the SDQ’s construct validity based on a 5-factor structure congruent with the measure’s five subscales [35]. The SDQ Total Difficulties Scale demonstrates strong concurrent validity with the Child Behavior Checklist total score (*r* = .70 – 0.87) and the Rutter parent questionnaire total deviance score (*r* = .76 − .88). In the present sample, internal consistency of the Externalizing scale was acceptable for the 2-4-year-old version (α = 0.73; *N* = 7) and the 4-10-year-old version (α = 0.70; *N* = 78). Externalizing scale internal consistency was lower for the 11-17-year-old version (α = 0.50; *N* = 24).

#### Parenting Locus of Control Scale

The Parenting Locus of Control Scale (PLOC; Campis et al., [36] is a 47-item self-report measure of parental locus of control. The PLOC scale measures five factors of parental locus of control: Parental Efficacy, Parental Responsibility, Parental Belief in Fate/Chance, Child Control of Parents’ Life, and Parental Control of Child’s Behavior. A higher overall score is indicative of a more external parental locus of control. The PLOC scale also demonstrated acceptable construct validity, as compared to the Internal External Scale (I-E) [36]. Internal consistency was adequate in this sample as well, with an overall Cronbach’s alpha reliability coefficient of 0.86. Cronbach’s alpha coefficients for the individual factors in this sample were 0.74 for Parental Efficacy, 0.81 for Parental Responsibility, 0.79 for Child Control of Parents’ Life, 0.61 for Parental Belief in Fate/Chance, and 0.81 for Parental Control of Child’s Behavior.

#### Brief Acceptability Measure

The Treatment Acceptability Rating Form – Revised (TARF-R) assesses the acceptability of parent-involved behavioral treatments [25]. The 20 items measure six components of treatment acceptability: perceived reasonableness, perceived effectiveness, side effects or disadvantages, disruptiveness, time required, and willingness to implement. Though the measure in its entirety has strong construct validity, Reimers et al. [25] found willingness to implement was the only component significantly correlated with parents’ actual implementation of behavior management strategies within one month (*r* = .53; *p* <. 01). At 3- and 6-month follow-ups, willingness to implement continued to be the strongest predictor of implementation, with correlation coefficients of 0.76 (*p* < .01) and 0.71 (*p* < .01), respectively. Thus, the three willingness to implement items from the TARF-R were used. A higher score on these summed items related to a greater willingness to use the strategy. The present sample demonstrated acceptable internal consistency reliability of TARF-R willingness items (α = 0.9).

### Procedure

Participants accessed the study on their own devices, presented via Qualtrics. Following informed consent, participants completed two screening questions. Those who confirmed their child demonstrated disruptive behaviors and they had not participated in behavioral interventions continued and completed the demographic questionnaire, SDQ, and PLOC. Parents then watched a set of videos explaining strategies commonly taught in PMT programs: child-directed interaction (CDI), effective commands, response cost, time-out, and differential attention. These interventions were selected to cover a large portion of the skills used in most PMT programs and to assess both proactive and reactive strategies. Videos were created for the purposes of this study and were 3-8-minutes in length and parents had the option to turn on captions if preferred. Following each video, parents rated their willingness to implement the strategy. Each video was presented on a separate screen from the TARF-R willingness questions and participants were not able to click forward to the TARF-R screen until the video was complete. After parents completed all videos and items, they had the option to enter their mailing or email address to receive a $50 gift card. Each strategy detailed in the videos is described below:

#### Child-Directed Interaction (CDI)

CDI, referred to in some PMT protocols as Special Time, is an intervention designed to increase positive interactions between parent and child. Parents designate a 5–15-minute period each day to spend engaging the child in play or an activity. During CDI, the parent interacts by praising, reflecting the child’s speech, imitating the child’s behavior, describing what the child is doing, and responding with enthusiasm. The parent avoids commands, questions, and criticisms. Minor disruptive behaviors are ignored and, if the child does not respond to a warning, major disruptive behaviors result in CDI ending early. This intervention is typically one of the first interventions presented to the parent during PMT and is designed to build the parent-child relationship rather than directly address disruptive behavior [37, 38].

#### Effective Commands

Parents are taught to use effective commands in PMT, which involve stating the command as a statement rather than a question, using a calm tone, minimizing the number of steps involved in the command, and keeping the command age appropriate. This antecedent strategy is designed to prevent disruptive behaviors by reducing the chance the child will interpret the command as optional, become dysregulated by complex commands or tone, or not be able to carry out the steps in the command [39].

#### Differential Attention

In differential attention interventions, adults selectively provide attention to desired behaviors. They simultaneously minimize attention to disruptive behaviors by ignoring and briefly withholding interaction from the child. As a result, the child learns to.

engage in behaviors that result in reinforcement [40]. Differential attention alters both the antecedent and consequence conditions surrounding the child’s disruptive behavior. Based on the two-part nature of this strategy, parents provided separate ratings of their willingness to implement positive attending, the proactive component of differential attention, and active ignoring, the reactive component of differential attention.

#### Time-Out

Time-out removes reinforcement when the child engages in more severe disruptive behaviors, such as aggression. Parents give the child time-out from reinforcement by moving them to a different location, stopping their activity, and withholding attention until the child is calm. The “time in” environment should be naturally reinforcing, with access to positive attention or activities, in order to make the time-out meaningful. This reactive consequence strategy is designed to respond to disruptive behavior and reduce the likelihood of it occurring in the future [41–43].

#### Response Cost

Response cost is a punishment strategy in which the child loses reinforcers when they engage in disruptive behavior. The amount of reinforcer lost and the behaviors that result in the punishment procedure are pre-determined and appropriate for the severity of the disruptive behavior. This reactive consequence strategy is designed to respond to disruptive behavior and reduce the likelihood of it occurring in the future [44].

## Results

To address the hypotheses, planned analyses included univariate correlation to identify the relation between PLOC and overall willingness to implement. Individual linear regression analyses were planned to assess the prediction of willingness to implement each strategy based on PLOC, while controlling for child gender, child age, and disruptive behavior severity. Hierarchical regression was planned to assess the predictive power of PLOC on overall willingness to implement, above the influence of child gender, child age, and behavior severity.

Descriptive statistics for the total willingness to implement score (sum of all willingness items), individual strategy willingness scores, total PLOC score, and SDQ Externalizing score are presented in Table 3. POMP (Percent of Maximum Possible) scores, or conversions of raw scores to a 100-point scale, are presented in Table 3 to aid in interpretation of raw scores. The maximum possible score for each strategy’s willingness to implement was 21, which indicates high willingness to use the strategy. Parents generally endorsed high rates of willingness to implement, with similar levels of willingness for each strategy, with the exception of Time Out. Mean willingness to implement Time Out was lower than mean willingness to implement the other strategies, and greater variability in responses was noted (M = 15.7; SD = 5.1).

**Table 3:** Descriptive statistics of key variables

Variable	Raw score		POMP* score
	M	SD	M SD
Parental Locus of Control Total Score	131.9	19.8	45.2 10.5
SDQ Externalizing Score Total Willingness to Implement Willingness: Proactive Strategies Willingness: Reactive Strategies Willingness: CDI Willingness: Effective Commands Willingness: Differential Attention – Positive Attending Willingness: Differential Attention – Active Ignoring Willingness: Time Out Willingness: Response Cost	10.1 107.1 54.72 52.38 17.8 18.3 18.7 18.1 15.7 18.6	2.4 14.2 6.84 8.85 2.9 2.7 2.5 3.0 5.1 3.2	50.3 12.0 84.1 11.9 84.7 12.7 80.3 16.4 82.0 16.3 84.7 15.2 87.3 14.0 83.9 16.4 70.6 28.3 86.4 17.7

_*Percent Of Maximum Possible_

Each variable was reviewed to check for statistical assumptions, including normality and linearity. Distributions were acceptable for all variables except for willingness to implement response cost, which was highly leptokurtic (5.99). A square root transformation reduced the kurtosis to an acceptable level (0.88). Skewness variables were negative for all willingness variables, which is consistent with previous findings that parents often find PMT acceptable [15, 29, 30].

Total willingness to implement was calculated by summing the willingness to implement score for each of the PMT strategies. Total willingness and PLOC were negatively correlated to a medium degree; *r* = − .32, *p* < .01. Higher PLOC scores, indicating greater externality of PLOC, were correlated with lower endorsement of willingness to implement PMT strategies as presented in a brief educational video. The relationships between PLOC and individual strategies were also assessed using regression analyses. Regression analyses were conducted separately for each strategy, as presented in Table 4 and 5. The combination of child age, child gender, SDQ Externalizing score, and PLOC predicted willingness to implement CDI to a small-to-medium degree (*F*(4,104) = 3.08, *p* < .05, adjusted *R*^*2*^ = 0.07). PLOC was a statistically significant predictor (*p* < .01), while child gender (*p* = .31), child age (*p* = .95), and SDQ Externalizing score (*p* = .35) were not. As willingness to implement CDI increased, a decrease in PLOC (indicating greater internality of PLOC) was observed.

Similarly, the combination of child age, child gender, SDQ Externalizing score, and PLOC predicted willingness to implement effective commands (*F*(4,104) = 2.95, *p* < .05, adjusted *R*^*2*^ = 0.07). In this model, PLOC was a statistically significant predictor (*p* < .01), while child gender (*p* = .82), child age (*p* = .96), and SDQ Externalizing score (*p* = .36) were not. Additionally, the combination of child age, child gender, SDQ Externalizing score, and PLOC predicted willingness to implement differential attention - positive attending (*F*(4,104) = 4.60, *p* < .01, adjusted *R*^*2*^ = 0.12). PLOC (*p* < .001) and child gender (*p* < .05) were statistically significant predictors of willingness to implement differential attention – positive attending, while child age (*p* = .50), and SDQ Externalizing score (*p* = .82) were not.

The combination of child age, child gender, SDQ Externalizing score, and PLOC as predictors of willingness to implement differential attention – ignoring resulted in a statistically insignificant model (*F*(4,104) = 0.64, *p* = .64, adjusted *R*^*2*^ = − 0.01) and no statistically significant predictors. While the combination of child age, child gender, SDQ Externalizing score, and PLOC predicted willingness to implement time out (*F*(4,104) = 2.91, *p* < .05, adjusted *R*^*2*^ = 0.03), none of these variables were independent statistically significant predictors of PLOC. Finally, the combination of child age, child gender, SDQ Externalizing score, and PLOC as predictors of willingness to implement response cost resulted in a statistically insignificant model (*F*(4,104) = 2.2, *p* = .07, adjusted *R*^*2*^ = 0.04), though PLOC was a statistically significant predictor (*p* < .01).

**Table 4:** Summary of regression analyses for PLOC predicting willingness to implement proactive strategies

	Willingness: CDI			Willingness: EC			Willingness: DA-PA		
Variable	B	SE B	β	B	SE B	β	B	SE B	β
Child Gender	− 0.59	0.58	− 0.10	− 0.12	0.54	− 0.02	-1.05*	0.48	− 0.20
Child Age	− 0.01	0.10	− 0.01	− 0.01	0.10	− 0.01	− 0.06	0.09	− 0.01
SDQ EXT	− 0.11	0.11	− 0.09	− 0.10	0.11	− 0.09	0.02	0.1	0.02
PLOC	− 0.04**	0.01	− 0.30	− 0.04**	0.01	− 0.31	− 0.04***	0.01	− 0.31
*R* ^*2*^	0.07			0.07			0.11		
*F*	3.08*			2.95*			4.60**		

_*p<.05; **p<.01; ***p<.001_

_CDI: Child−Directed Interaction; EC: Effective Commands; DA−P: Differential Attention−Positive Attending; SDQ EXT: SDQ Externalizing Score_

**Table 5:** Summary of regression analyses for PLOC predicting willingness to implement reactive strategies

	Willingness: DA-AI			Willingness: TO			Willingness: RC		
Variable	B	SE B	β	B	SE B	β	B	SE B	β
Child Gender	0.03	0.61	− 0.01	− 0.22	1.00	− 0.02	− 0.17	0.15	− 0.1
Child Age	− 0.05	− 0.05	− 0.04	− 0.30	0.18	− 0.16	0.04	0.03	0.15
SDQ EXT	− 0.04	− 0.04	− 0.03	0.37	0.20	0.17	0.01	0.03	0.03
PLOC	− 0.02	− 0.02	− 0.14	− 0.04	0.02	− 0.17	− 0.01**	0.01	− 0.26
*R* ^*2*^	− 0.01			0.03			0.04		
*F*	0.63			2.91*			2.2		

_*p<.05; **p<.01; ***p<.001_

_DA−AI: Differential Attention−Active Ignoring; TO: Time Out; RC: Response Cost; SDQ EXT: SDQ Externalizing Score_

To investigate the predictive power of PLOC on total willingness to implement all six strategies, after controlling for child gender, child age, and disruptive behavior severity, a hierarchical linear regression was conducted. When child gender and age were entered into the model alone, these variables did not significantly predict willingness; *F*(2,106) = 1.08, *p* = .34, adjusted *R*^*2*^ = 0.0014. The addition of disruptive behavior severity (SDQ Externalizing score) to the model did not yield an increase in adjusted *R*^*2*^; *F*(3,105) = 0.74, *p* = .53, adjusted *R*^*2*^ = − 0.01. The addition of PLOC to the model improved prediction of willingness, with a small effect; *F*(4,104) = 3.39, *p* < .01, adjusted *R*^*2*^ = 0.08. The final model accounts for 8% of the predicted variance in willingness to implement PMT strategies. The values presented in Table 6 indicate the contribution of each variable to willingness to implement, when child age and gender, SDQ Externalizing score, and PLOC are entered into the model together.

**Table 6:** Hierarchical multiple regression analysis summary predicting willingness to implement from child age, child gender, SDQ externalizing score, and PLOC total score

Variable	*B*	*SEB*	*βR* ^*2*^	*ΔR* ^*2*^
Step 1			0.001	0.001
Child Gender	-1.89	2.87	-0.06	
Child Age	-0.57	0.5	-0.11	
Step 2			-0.007	-0.008
Child Gender	-1.95	2.89	-0.07	
Child Age	-0.56	0.5	-0.11	
SDQ Externalizing Score	0.15	0.57	0.03	
Step 3			0.08	0.08
Child Gender	-2.7	2.7	-0.09	
Child Age	-0.26	0.49	-0.05	
SDQ Externalizing Score	0.19	0.55	0.03	
PLOC Total Score	− .22**	0.07	− .31	

_*p<.05; **p<.001_

## Discussion

This study sought to identify the relationship between parental locus of control and willingness to implement PMT strategies and add to the understanding of parent cognitions as influencers of parent engagement in PMT. Additionally, this study investigated this relationship for individual components of PMT, comparing participants’ willingness to implement strategies used proactively to prevent disruptive behavior and those used reactively in response to disruptive behavior. Ultimately, by attending to parent cognitions such as PLOC and considering variation in parents’ response to each strategy, researchers and clinicians can better personalize PMT, facilitate increased parent engagement, and, in turn, improve child outcomes [11, 17].

Results indicate PLOC and overall willingness to implement PMT strategies have a significant inverse relationship, as hypothesized. The more parents express an internal PLOC, the greater their willingness to implement PMT strategies. Inversely, the more parents express external PLOC, the lower their willingness to implement PMT strategies. Additionally, results indicated PLOC is a small but significant predictor of overall willingness to implement, when controlling for child gender, age, and disruptive behavior severity.

PLOC also predicted willingness to implement proactive PMT strategies (CDI, Effective Commands, Differential Attention – Positive Attending), when child age and gender and disruptive behavior severity were held constant. Internal PLOC predicted greater willingness to implement these strategies. Parents who endorse external PLOC are more likely to endorse lower willingness implement proactive strategies. This finding fits the understanding that parents with external PLOC are less likely to believe their parenting will impact their child’s behavior [20]. We theorize this belief results in lower willingness to attempt preventative strategies. The relationship between PLOC and willingness to implement proactive strategies is notable because proactive strategies are presented first in most manualized PMT programs [2]. Thus, the first strategies presented in PMT are perhaps the least compatible with the beliefs of a parent with external PLOC. This could contribute to early treatment dropout or low treatment engagement.

PLOC did not predict willingness to implement two of the three reactive strategies (differential attention – active ignoring and time out). While PLOC did predict willingness to implement the third reactive strategy, response cost, the degree of this predictive relationship was smaller than that of the proactive strategies. Reactive PMT strategies may be adequately aligned with both internal and external PLOC-related beliefs, for different reasons. For parents with internal PLOC, their willingness to use these strategies may be supported by their parental self-efficacy. For parents with external PLOC, reactive strategies may be well-aligned with their existing practices, as those with external PLOC are more likely to use authoritarian, permissive, or inconsistent parenting approaches and emphasize reactive responses to behavior [20, 21].

### Implications and Future Directions

These findings have useful implications for clinicians seeking to increase parent engagement in PMT. While effect sizes were small to medium, this study provides support for the relationship between PLOC and willingness to implement strategies. Parents with external PLOC are more likely to be ambivalent toward treatment and hold maladaptive beliefs about their inability to change their child’s behavior or use the strategies effectively. In order to address limited willingness, clinicians must first understand the parent’s PLOC. This can be assessed during the intake or referral process using a formal measure such as the Parental Locus of Control scale [26]. Alternatively, clinicians may prefer to incorporate questions related to PLOC into their clinical interview. Should clinicians find the 47-item PLOC scale or interview questions about all five PLOC components longer than is necessary or realistic for their setting, it may be sufficient to focus on beliefs related to parental efficacy and the role of fate and chance. These PLOC subscale components were more highly correlated to willingness to implement than the other subscale constructs.

When PLOC is briefly assessed and parents with external PLOC are identified, clinicians can adjust their treatment plan accordingly to maximize the likelihood of parent engagement. Such parents may benefit from early efforts to address their misalignment with the initial strategies. For each strategy, clinicians should ensure parents understand the treatment rationale and how it applies to their child’s behavioral goals, and whether parents are willing to attempt it and provide honest feedback to the clinician. Motivational interviewing strategies or explicit identification of the fit between treatment goals and family values could increase parent buy-in [45]. Psychoeducation could address misconceptions about disruptive behavior and treatment, such as the parental belief that disruptive behavior cannot be prevented or occurs entirely at random, or that their child’s disruptive behavior is age-appropriate or will improve on its own. McCabe et al.’s [17] PersIn model demonstrated a similar approach by collecting data on parents’ causal attributions and treatment expectancies and using the information to drive individual modifications to Parent-Child Interaction Therapy (PCIT), a form of PMT.

For some parents, particularly those who do not respond to individualized psychoeducation and whose limited compliance with proactive strategies make them susceptible to drop out, more significant adjustments to the treatment plan may be necessary. The initial strategies presented in PMT programs are intended to build the parent-child relationship, reinforce positive behavior, and prevent disruptive behavior. Reactive strategies follow in the latter half of treatment, based on the rationale that the proactive strategies increase the effectiveness of consequence strategies and prevent the frequency with which they are needed [2]. While this rationale is sound and works for many families, it offers limited benefit to parents who drop out after one session. Future research could investigate the value of offering reactive strategies earlier in treatment to parents with external PLOC, in order to gain parent buy-in. This may also increase parental self-efficacy if they experience success with the strategy. Miller and Prinz [23] utilized a similar rationale by adjusting the treatment format (e.g., parent-only, child-only, parent and child) according to parents’ causal attributions. When the treatment format and parental attributions matched, parents were less likely to drop out.

When individualizing treatment, clinicians should consider the impact of PLOC on each treatment component. As highlighted by the varying relationships between willingness to implement and PLOC among PMT strategies, low willingness for one strategy may not extend to other strategies or to PMT overall. Engagement in one strategy does not mean the parent will remain engaged in all strategies. Clinicians should assess acceptability before and throughout treatment. Clinicians can accomplish this through brief check-ins to assess parent willingness and related beliefs as each new strategy is presented and attempted. Providers could also take a more systematic approach by presenting a brief overview of each strategy, or a video similar to those used in the present study, and formally assessing parental willingness to implement using the TARF-R willingness items. Though assessing willingness via videos and rating scale items requires more preparation from the clinician, it could be completed online by parents who are on waitlists for treatment and allow for efficient, individualized treatment planning.

Similarly, in investigating the role of parent beliefs in treatment acceptability and engagement, future research should acknowledge the composite makeup of PMT and analyze components individually, as they may be differentially impacted by parent beliefs. While parental beliefs are relevant to all stages of treatment, it is particularly important to understand the impact of parent characteristics on strategies taught early in treatment, as treatment drop out can occur after a single session [10]. Research attending to the acceptability of individual components of PMT is more likely to guide clinicians in providing treatment that is responsive to changing needs of parents throughout each session, as well as the treatment arc.

Further, future studies should investigate clinical use of these findings, including the feasibility and acceptability of measurement of PLOC during the referral, assessment, or intake process. The influence of PLOC on actual parent engagement and treatment retention, in addition to reported willingness to implement, should also be researched. Understanding of the practical benefit of PLOC assessment and PLOC-based treatment adjustments will contribute to clinicians’ willingness to incorporate these findings into their practice. Improving parental engagement and retention has the potential to improve more than child outcomes; it is conceivable that such improvements can decrease treatment costs and lower clinician stress.

### Strengths and Limitations

The methodology of this study presents several implications for interpretation and generalizability of the results. First, participants were recruited irrespective of their current help-seeking status or intention to change their parenting behavior. This provided a sample representative of the continuum of readiness for change observed in clinical settings, benefitting generalizability [46]. However, help-seeking status and readiness for change may be influential predictor variables not quantified in this study. Parents with less help-seeking intention may have been less likely to perceive strategies as necessary or reasonable, and thus less likely to endorse willingness to implement [46]. Similarly, disruptive behavior severity was measured, but qualitative characteristics of the behaviors were not. Future studies may consider the role of disruptive behavior type (e.g. aggressive behavior vs. non-aggressive behavior) in parent treatment-related cognitions and engagement.

The online presentation of the study is both a strength and limitation. This format was efficient, cost-effective, and allowed for the assessment of parents’ immediate response to PMT strategies. This provides real-time data about internal reactions to PMT strategies when they are presented, rather than parents’ retrospective accounts of their reaction. The online presentation does create uncertainty about parent’s level of attention to the videos. However, research indicates data gathered online tend to have similar, or better, quality when compared to in-person data [47]. Additionally, measures were taken to reduce the likelihood of bot responses, duplicate responders, or rushed responding. Still, participants’ attention to the videos and questions may have varied, resulting in a potential confounding variable.

Finally, while a variety of demographic identities were represented in the sample, the sample included an overrepresentation of white and high SES parents, which impacts generalizability to clinical populations. The sample also overrepresented female caregivers as compared to the general population, though this is consistent with the rates of mothers attending PMT, compared to fathers [48]. Limited data on male caregivers in PMT makes it difficult to determine influencers of their engagement. Future analyses should control for these caregiver demographic characteristics on willingness to implement PMT strategies.

## Summary

This study offers support for the relevance of individual parental beliefs in PMT treatment acceptability and highlights the potential for PLOC to guide the personalization of PMT. Further research is needed to understand the complex interactions between PLOC, related parental beliefs and values, parent treatment intentions, and actual treatment engagement. As we grow our understanding of the nuances of parental beliefs and their role in treatment, our capacity to adapt and expand the reach of PMT to more families will grow as well.

## Data Availability

All data supporting the findings of this study are available from the authors upon reasonable request.
